# ID 32 - Segurança e Eficácia da Terapia de Estimulação Cognitiva (*Cognitive Stimulation Therapy* – CST) para Pacientes com Doença de Alzheimer Leve a Moderada

**DOI:** 10.5327/2237-9622.2025.v34s1.32

**Published:** 2025-11-25

**Authors:** Valter Paulo Neves Miranda, Maria Helha Fernandes do Nascimento, Andreia Rosa Borgesa, Annelissa Andrade Virgínio de Oliveira, Jeamile Lima Bezerra, Tassiane Cristine Santos de Paula

**Keywords:** doença de Alzheimer, demência, cognição, qualidade de vida, atividades diárias, revisão sistemática

## Abstract

**Introdução:**

A doença de Alzheimer (DA) é a causa mais comum de demência, representando cerca de 60% a 80% dos casos (Nice, 2018). A Terapia de Estimulação Cognitiva (CST) é uma intervenção não farmacológica que visa melhorar a cognição, a comunicação e a qualidade das atividades de vida diária (AVD) de indivíduos com DA (Yuill, Hollis, 2011). O objetivo do estudo foi avaliar a segurança e a eficácia da CST para pessoas com DA leve a moderada para a cognição, qualidade de vida e atividade de vida diária.

**Método:**

Revisão sistemática com metanálise de ensaios clínicos randomizados controlados (ECRs). A busca dos estudos foi realizada nas bases de dados PubMed, Embase, Cochrane Library e LILACS. O processo de seleção foi feito no software Rayyan e a avaliação do risco de viés na ferramenta RoB 2.0. Foram elegíveis os estudos com as pessoas de ambos os sexos e que apresentavam diagnóstico de demência e DA leve a moderada, segundo os critérios do DSM-5. Foram extraídos dados da linha de base e após a intervenção entre os grupos que receberam a intervenção da CST e o grupo controle (TAU). O software Review Manager versão 5.4 foi usado para a metanálise. Foram extraídos os da diferença de média (DM) e diferença de média padronizada (DMP) dos desfechos. O software Webplotdigitizer foi usado na extração de dados das figuras. A certeza no conjunto final da evidência foi avaliada por meio da abordagem (GRADE).

**Resultados:**

Um total de 563 referências foram avaliadas pela triagem de títulos e resumos, das quais 52 foram selecionadas para a leitura completa. Por fim, nove ECR foram incluídos, com total de 923 participantes. Em sete estudos, a CST foi realizada conforme o manual proposto por Spector et al. (2003). Não foram encontrados efeitos adversos graves. A metanálise evidenciou que a CST foi benéfica na redução da velocidade do declínio cognitivo (DMP: 0,50, IC 95%: 0,16-0,83) e na melhora da QV (DM: 1,45, IC 95% 0,82-2,07). Não houve melhora nas AVDs (DMP: 0,23, IC 95%: −0.07-0,52).

**Conclusão:**

Ao avaliar o risco de viés, identificou-se que dois estudos apresentaram "baixo risco" geral, enquanto os demais estudos levantaram "algumas preocupações". Na avaliação do conjunto das evidências com base na ferramenta GRADE, a certeza dos efeitos da CST sobre a cognição foi considerada baixa. Com isso, essas considerações foram cruciais para a avaliação e a interpretação cuidadosa dos resultados deste estudo, ao generalizar as conclusões para a prática clínica. Cinco dos nove estudos demonstraram melhora na função cognitiva. No entanto, apenas um estudo observou melhoria a curto e longo prazo. Nesse caso, o benefício envolveu tanto a função da memória como a de linguagem. Esse achado indica que, para além da memória a CST pode contribuir em outros aspectos da cognição, e com isso, impactar na saúde das pessoas com DA. A CST pode contribuir para a melhora das habilidades sociais, pois geralmente é realizada em grupo, promovendo a interação entre os participantes, o que favorece aspectos relacionados à saúde mental e ao bem-estar geral. Conclui-se que a CST foi eficaz na redução do declínio cognitivo e na qualidade de vida de pacientes com DA leve e moderada. Os resultados encontrados precisam ser interpretados com cautela, pois a certeza das evidências relativas aos efeitos do tratamento da CST foi classificada como baixa, em relação ao desfecho primário cognição.

